# Colorectal Adenomas Contain Multiple Somatic Mutations That Do Not Coincide with Synchronous Adenocarcinoma Specimens

**DOI:** 10.1371/journal.pone.0119946

**Published:** 2015-03-16

**Authors:** José P. Vaqué, Nerea Martínez, Ignacio Varela, Fidel Fernández, Marta Mayorga, Sophia Derdak, Sergi Beltrán, Thaidy Moreno, Carmen Almaraz, Gonzalo De las Heras, Mónica Bayés, Ivo Gut, Javier Crespo, Miguel A. Piris

**Affiliations:** 1 Cancer Genomics Group, IDIVAL, Instituto de Investigación Marqués de Valdecilla, Santander, Spain; 2 IBBTEC-UC-CSIC-SODERCAN Instituto de Biomedicina y Biotecnología de Cantabria, Santander, Spain; 3 Department of Pathology, Hospital Universitario Marqués de Valdecilla, Santander, Spain; 4 Centro Nacional de Análisis Genómico, CNAG, Barcelona, Spain; 5 Gastroenterology and Hepatology Unit, Hospital Universitario Marqués de Valdecilla, Santander, Spain; 6 Infection, Immunity and Digestive Pathology Group, IFIMAV, Santander, Spain; Harvard Medical School, UNITED STATES

## Abstract

We have performed a comparative ultrasequencing study of multiple colorectal lesions obtained simultaneously from four patients. Our data show that benign lesions (adenomatous or hyperplastic polyps) contain a high mutational load. Additionally multiple synchronous colorectal lesions show non overlapping mutational signatures highlighting the degree of heterogeneity between multiple specimens in the same patient. Observations in these cases imply that considering not only the number of mutations but an effective oncogenic combination of mutations can determine the malignant progression of colorectal lesions.

## Introduction

Our current understanding of colorectal cancer assumes that its pathogenesis includes a progressive accumulation of genomic changes at multiple stages. Thus, initiating events, such as driver mutations affecting APC or KRAS genes, are followed by additional alterations in specific genes such as p16 and p53 [1] and signalling pathways including WNT, MAPK, GNAS or TGFB that, over time, will shape the genomic conditions that drive a pre-malignant lesion towards cancer [2–4]. Thus, premalignant lesions such as colorectal adenomas feature mutational events in APC, BRAF, KRAS and other genes [2, 5]. As the disease progresses, colorectal adenocarcinoma specimens can also accumulate mutations in genes such as p53 and FBXW7 as well as in MAPK, TGFB, PI3K and DNA mismatch-repair pathways [3]. However, the question of whether somatic mutations accumulate in the adenoma-carcinoma sequence in the same patient remains to be investigated.

Here we have sequenced whole exomes of multiple lesions in four non-MSI colorectal cancer patients corresponding to different adenoma and adenocarcinoma specimen samples taken during the same endoscopic procedure. Our first finding was that adenomas contained a large number of mutations that, in general were reduced but still comparable, with the frequency found in colorectal cancer samples. Additionally, different adenoma lesions within the same patient were strikingly heterogeneous. Analysis of the mutation frequency also showed that a large majority of the mutations found in adenoma samples were subclonal, and probably passenger mutation events.

## Results and Discussion

**Fig 1:**
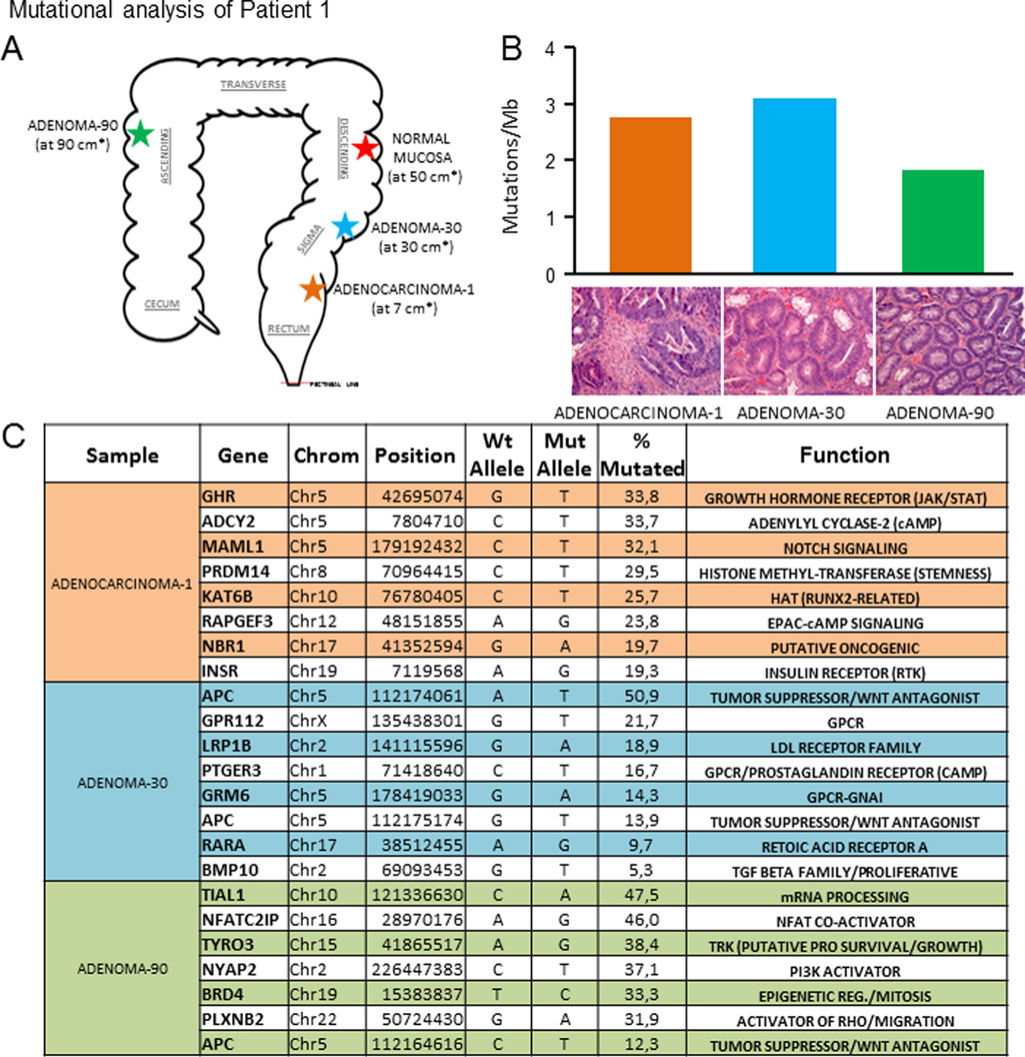
Mutation analysis of patient 1. A) Scheme showing an approximate representation of the location of each lesion analysed. The distance (*, in cms) from the pectineal line (red dots) is shown. B) Mutational index (number of mutations/Mb) found in the indicated sample from the primary NGS analysis. H&E pictures are representative of each lesion studied by NGS. C) Validated mutations found in a secondary targeted NGS analysis of the indicated samples. Chrom: chromosome; % mutated: percentage of mutant nucleotides found in the corresponding gene within the same sample.

**Fig 2:**
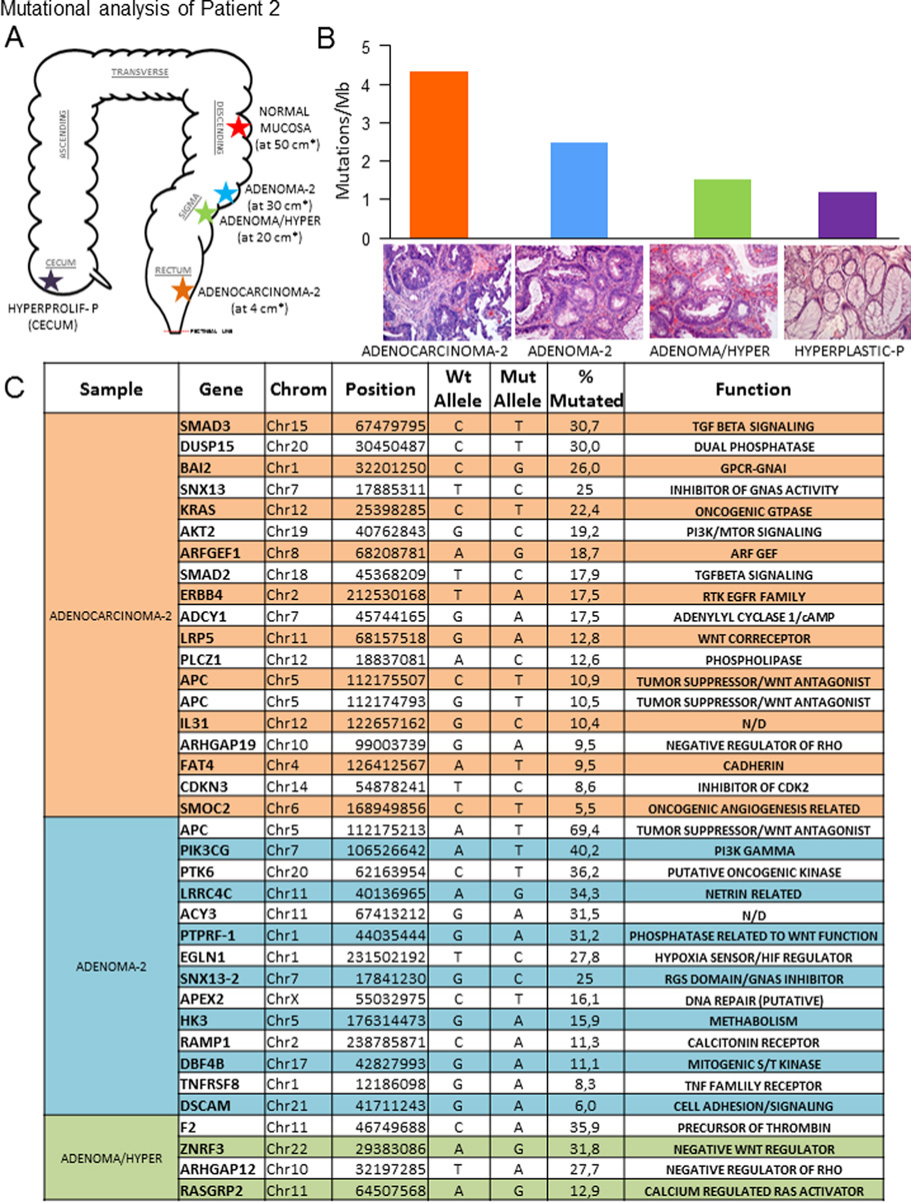
Mutation analysis of patient 2. A) Scheme showing an approximated representation of the location of each lesion analysed. The distance (*, in cms) from the pectineal line (red dots) is shown. B) Mutational index (number of mutations/Mb) found in the indicated sample from the primary NGS analysis. H&E pictures are representative of each lesion studied by NGS. C) Validated mutations found in a secondary targeted NGS analysis of the indicated samples. Chrom: chromosome; % mutated: percentage of mutant nucleotide found in the corresponding gene within the same sample.

**Fig 3:**
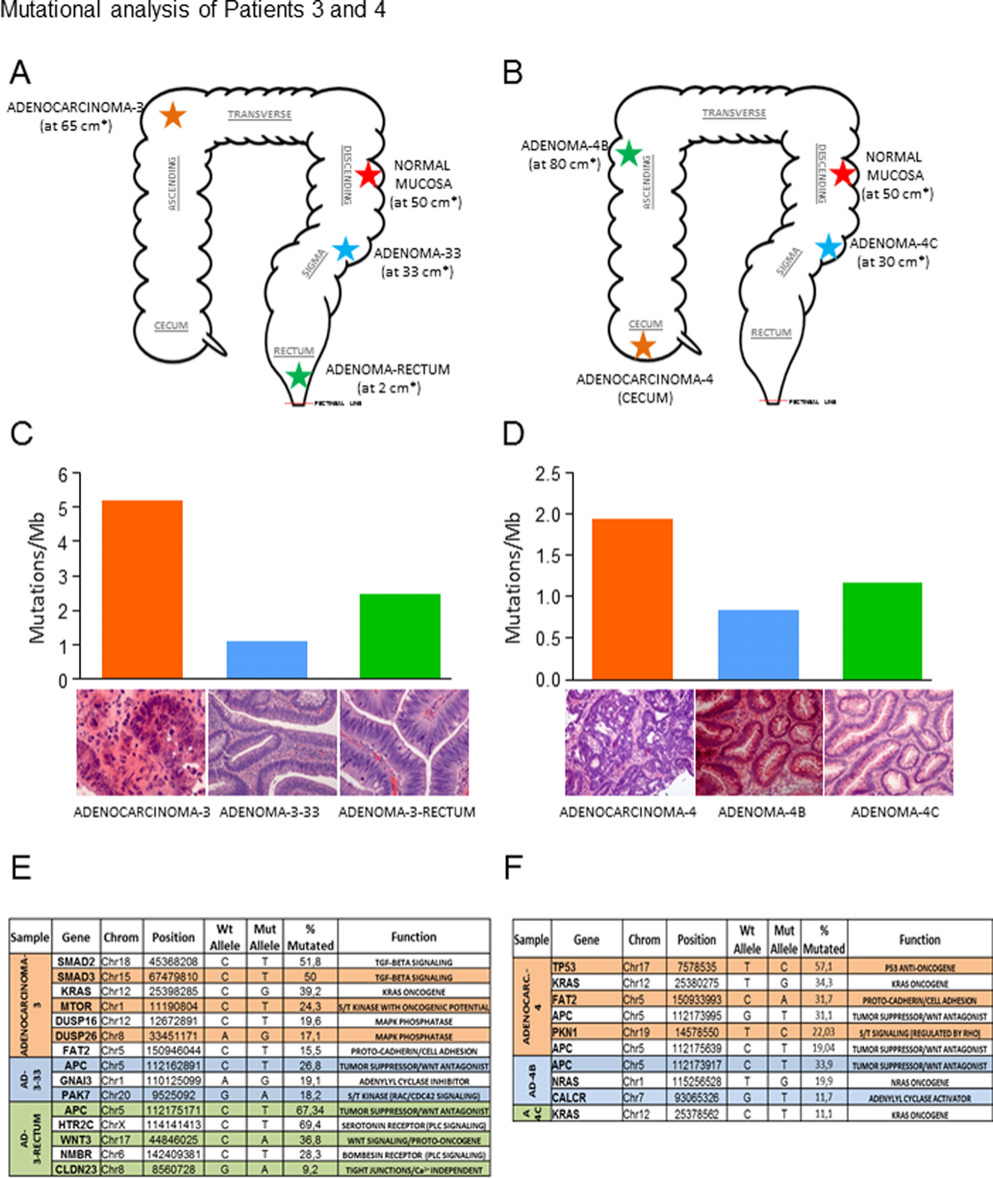
Mutation analyses of patients 3 and 4. Scheme showing an approximated representation of the location of each lesion analysed in patient-3 (A) and patient-4 (B). The distance (*, in cms) from the pectineal line (red dots) is shown. C), D) Mutational index (number of mutations/Mb) found in the indicated sample from the primary NGS analysis. H&E pictures are representative of each lesion studied by NGS. Tables below show a selection of genes with oncogenic potential found mutated in the primary analyses of patient-3 (E) and patient 4 (F). Chrom: chromosome; % mutated: percentage of mutant nucleotide found in the corresponding gene within the same sample.

**Fig 4:**
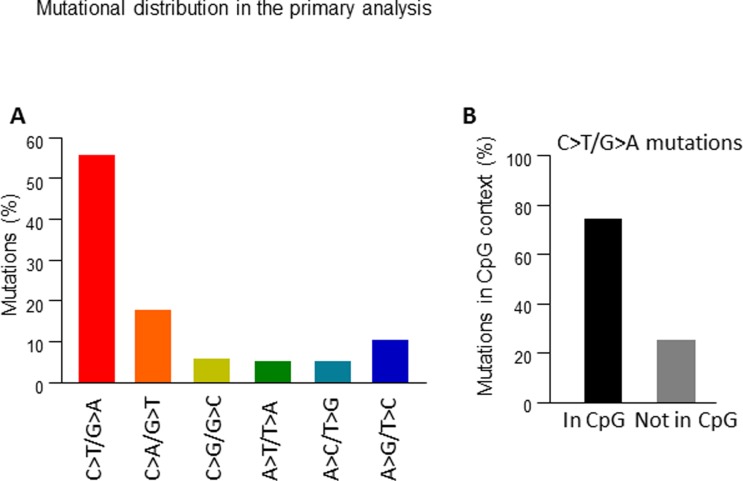
Distribution of mutations detected in the primary analysis. A). Percentage of the indicated mutations detected in the primary analysis. B) Percentage of mutations in CpG dimers.

We characterized the genomic variants in a series of untreated colorectal lesions that included adenocarcinomas, adenomas and hyperproliferative polyps taken simultaneously by endoscopic resection, along with normal mucosa, which was used as a control (S1 Table). The topologies of the lesions of each patient are shown in Figs. 1a, 2a, 3a and 3b and the clinical characteristics are summarised in Table 1. We generated two paired-end 75-bp whole exome sequencing libraries and sequenced them using an Illumina HiSeq2000 instrument, which allowed us to map an average of ~102 million reads per sample. Under these conditions, the mean coverage of the target sequenced was 99X (78X-141X) with a mean of 92.1% (89.8–95.9) of targeted bases with at least 15X coverage (S1 Table). Somatic variants were identified using the SAMtools suite. Additionally, we used RAMSES software [6] to call potential mutations showing minimum independent multi-aligner evidence that enabled us to identify subclonal variants present in at least 5% of the reads. We also performed a secondary analysis in a selection of genes with known biological activity that confirmed specific mutations in up to 76.5% of those genes with a mutational percentage above 15% in each sample of our primary analysis (Figs. 1b and 2b and S1 Fig. and S2 Table). Using the data obtained in our primary analysis and aligned with previous observations in colorectal lesions [5], we observed that most mutations were C>T/G>A changes that occurred in CpG in up to 75% of the cases (Fig. 4, and S5 Table). In addition, we reproduced these results using the validated data from the secondary analysis (S2 Fig.). A detailed description of the main findings is included in table 2 and S1–S5 Tables. We decided to focus on those alterations that could potentially induce changes in the expression or activity of the proteins including amino acid changing or truncating mutations. Analysing their incidence, we found that most but not all benign lesions (adenoma or hyperproliferative polyp) contained less genomic alterations than the colorectal cancer specimens (Figs. 1b, 2b, 3a and 3b and table 2); a mutational rate similar to that described by the TCGA network for the non-hypermutated colorectal adenocarcinoma samples [3]. Using this approach we were able to detect one or multiple distinct gene alterations affecting APC in 6 of the 8 adenomas analysed, thereby underlining the relevance of the APC gene inactivation in the genesis of colorectal adenomas. In the same line of evidence, we observed that these benign lesions lacked mutations in genes or pathways considered essential in colorectal cancer [3], with the possible exception of PIK3CG in the adenoma-2 case (Fig. 2c) or KRAS and NRAS mutations found in adenomas-4B and 4C (Fig. 3f). On the other hand, we noticed that a number of mutations found in the adenocarcinomas affected oncogenes such as GHR and INSR (Fig. 1c) or KRAS and ERBB4 (Fig. 2c). These are well known for their ability to activate MAPK signalling. We were able to detect them alongside other somatic mutations affecting SMAD genes (TGFB signalling, Fig. 2c and Fig. 3e) or adenylyl cyclases such as ADCY2 (Fig. 1c) and ADCY1 (Fig. 2c) that participate in the COX2-PGE2-PR-GNAS signalling axis (reviewed in [7]). When comparing the mutational spectrum of the multiple samples from the same patient, we did not find a single recurrent mutation, which in addition to the multiple and non-recurrent alterations found in APC, suggests an independent origin of the multiple adenomas and adenocarcinoma in the same patient. In this respect, we could detect individual lesions like for example adenoma-30 (Fig. 1), carrying different mutations in APC detected at different percentages (14% and 51%). This may reflect a degree of subclonal activity that is not exclusive to adenomas, since adenocarcinoma-2 (Fig. 2) also harboured two distinct APC mutations in 10.9% and 10.4% of the alleles read. Moreover, our observations (aligned with those found in [5]), seem to suggest that colorectal adenomas, independently of their size or degree of dysplasia, and even hyperplastic polyps, (both with reduced potential to make progress towards cancer), still feature a relatively high number of subclonal mutations combined into inefficient non-carcinogenic signatures. Thus, early steps of colorectal cancer could be characterised by highly dynamic genetic changes until an efficient neoplastic signature, giving rise to an infiltrating carcinoma, is generated. Due to the limited number of patients analysed we cannot yet generalize whether all benign lesions carry a high mutational load. This may also apply to the observation that mutations found in adenomas do not coincide with those found in synchronous adenocarcinoma specimens in the same patient, a finding that is supported by data from other laboratories [5]. The individual characterisation of these precise mutational signatures controlling tumour dynamics at specific stages of the disease may serve in the near future as an indicator for the development of specific combination therapies.

**Table 1:** Clinical description of the samples analysed.

PATIENT	SEX	AGE	SAMPLE	SAMPLE NAME	DIAGNOSTIC	GRADE OF DYSPLASIA	SIZE (CMS)	MACROSCOPIC DESCRIPTION	LOCALIZATION (FROM PECTINEAL LINE)
1	FEMALE	82	N1E073	normal mucosa-1	normal mucosa	N/A	N/A	healthy mucosa without any macro- or microscopic alteration	50 cm (descendent colon)
1	FEMALE	82	N1E074	Adenoma-30	adenomatous polyp	Tubular adenoma with moderate dysplasia	0,8	Semi-pedunculated polyp	30 cm (sigma)
1	FEMALE	82	N1E075	Adenoma-90	adenomatous polyp	Tubular adenoma showing moderate dysplasia with superficial focal severe dysplasia	0,8	Semi-pedunculated polyp	90 cm (ascendent colon)
1	FEMALE	82	N1E076	Adenocarcinoma-1	adenocarcinoma: poorly differentiated	N/A	5 (length)	stenotic and ulcerated circumferential mass	7 cm (rectum)
2	MALE	82	N2E079	normal mucosa-2	normal mucosa	N/A	N/A	healthy mucosa without any macro- or microscopic alteration	50 cm (descendent colon)
2	MALE	82	N2E069	hyperplastic polyp-2	hyperplastic polyp	N/A	0,3	sessile polyp	cecum
2	MALE	82	N2E092	Adenoma/Hyper-2	adenomatous polyp with hyperplastic mucosa	Tubular adenoma mostly showing mild dysplasia with focal moderate dysplasia	0,2	sessile polyp	20 cm (sigma)
2	MALE	82	N2E070	Adenoma-2	adenomatous polyp	Tubular adenoma mostly showing mild dysplasia with focal moderate dysplasia	0,5	sessile polyp	30 cm (descendent colon)
2	MALE	82	N2E072	Adenocarcinoma-2	adenocarcinoma: Well differentiated	N/A	6 (length)	ulcerated, circumferencial and friable mass (3/4ths of the rectal lumen)	4 cm (rectum)
3	MALE	71	N3J876	normal mucosa-3	normal mucosa	N/A	N/A	healthy mucosa without any macro- or microscopic alteration	50 cm (descendent colon)
3	MALE	71	N3J874	Adenoma-rectum	adenomatous polyp	Tubular adenoma mostly showing focal severe dysplasia	0,5	pedunculated polyp	2 cm (rectum)
3	MALE	71	N3J873	Adenoma-33	adenomatous polyp	Tubular adenoma mostly showing mild dysplasia	1	pedunculated polyp	33 cm (sigma: from anal margin)
3	MALE	71	N3J872	Adenocarcinoma-3	adenocarcinoma	N/A	5 (length)	stenotic and ulcerated circumferential mass	65 cm (hepatic angle)
4	MALE	62	N4J881	normal mucosa-4	normal mucosa	N/A	N/A	healthy mucosa without any macro- or microscopic alteration	50 cm (descendent colon)
4	MALE	62	N4J878	Adenoma-4B	adenomatous polyp	Tubular adenoma mostly showing mild dysplasia	0,8	Semi-pedunculated polyp	80 cm (ascendent colon)
4	MALE	62	N4J879	Adenoma-4C	adenomatous polyp	Tubular adenoma mostly showing mild dysplasia	0,6	Semi-pedunculated polyp	30 cm
4	MALE	62	N4J877	Adenocarcinoma-4	adenocarcinoma	N/A	8	Ulcerated circumferential mass. Occupies 1/2 of the rectal lumen	cecum

**Table 2:** Number of unique amino-acid changing mutations found in the primary analysis.

PATIENT	PATIENT SAMPLE	DATA SOURCE	MUTs*	MUT. INDEX	MUTs INVERSE ANALISYS**	MUT. INDEX (N)
1	N1E074	Adenoma-90 vs. normal mucosa-1	56	1,87	2	0,07
1	N1E075	Adenoma-30 vs. normal mucosa-1	93	3,10	3	0,10
1	N1E076	Adenocarcinoma 1 vs. normal mucosa-1	84	2,80	4	0,13
2	N2E069	Hyperplastic-P vs. normal mucosa-2	35	1,17	2	0,07
2	N2E092	Adenoma/Hyper vs. normal mucosa-2	45	1,50	6	0,20
2	N2E070	Adenoma 2 vs. normal mucosa-2	74	2,47	4	0,13
2	N2E072	Adenocarcinoma 2 vs. normal mucosa-2	130	4,33	4	0,13
3	N3J874	Adenoma-rectum vs normal mucosa-3	74	2,47	9	0,30
3	N3J885	Adenoma-33 vs normal mucosa-3	33	1,10	7	0,23
3	N3J872	Adenocarcinoma-3 vs normal mucosa-3	155	5,17	14	0,47
4	N4J878	Adenoma-4B vs normal mucosa-4	33	1,10	5	0,17
4	N4J879	Adenoma-4C vs normal mucosa-4	25	0,83	8	0,27
4	N4J877	Adenocarcinoma-4 vs normal mucosa-4	58	1,93	9	0,30

MUTs*: Number of amino-acid changing mutations in each lesion vs. normal mucosa, MUT. INDEX: Number of mutations/Mb (Exome) in the colorectal lesion, MUTs INVERSE ANALYSIS**: Number of amino-acid changing mutations found in normal mucosa vs. each lesion, MUT. INDEX (N): Number of mutations/Mb (Exome) in normal mucosa.

## Materials and Methods

### Ethics statement

All human samples used in this study were collected under a written informed consent form that was appropriately signed and authorized by each patient and the doctor(s) involved and approved by the CEIC (Comité Ético de Investigación Clínica, Cantabria). We kept the original records under specific restrictive conditions to fulfil the current legal requirements. All processes were conducted and approved following the specific recommendations of the CEIC.

### Patients and samples

Nine freshly frozen colorectal samples taken from two previously untreated patients by endoscopic resection were selected for whole exome sequencing. Samples from Patient 1 (Fig. 1) consisted of: a) normal mucosa, b) adenomatous polyp (30 cm), c) adenomatous polyp (90 cm) and d) adenocarcinoma. Samples from Patient 2 (Fig. 2) consisted of: a) normal mucosa, b) hyperplastic polyp, c) adenomatous polyp, d) adenomatous polyp and e) adenocarcinoma. Further information is provided in S1 and S5 Tables. All cases were reviewed by a panel of three pathology specialists and lesions were graded following standard criteria [8].

### Genomic DNA extraction, quantification, exome enrichment and sequencing

Purified genomic DNA (3 μg) was extracted from snap-frozen (fresh) samples using standard procedures. Briefly, PBS-washed samples, centrifuged and lysed using “Tissue and cell lysis solution” buffer for the MasterPure kit, complemented by proteinase K (5 μl/100 μl buffer) (Epicenter), shaking overnight at 56°C. DNA was extracted using phenol/chloroform/isoamyl alcohol (in proportions of 25:24:1, respectively) in a fast Lock Gel Light Eppendorf tube (Eppendorf), then washed and precipitated. Genomic DNA was quantified using a Qubit ds DNA BR assay kit and a Qubit 2.0 fluorometer (Invitrogen) following the manufacturer’s instructions. Genomic DNA (3 μg) was then enriched in each case for protein coding sequences using the in-solution exome capture SureSelect Human All Exon 50 Mb kit (Agilent Technologies), following the manufacturer’s protocol. The captured targets were subjected to massively parallel sequencing using the Illumina HiSeq 2000 Analyzer (Illumina) with the paired-end 2 × 75 bp read option, in accordance with the manufacturer’s instructions. Exome capture and massively parallel sequencing were performed at the Spanish National Genome Analysis Centre (CNAG, Barcelona, Spain). The raw data from this study have been deposited in the NIH Short Read Archive (SRA) under accession number SRP040626.

### Sequence mapping and identification of tumour variants

These methods have been described elsewhere [6]. Briefly, base calling and quality control were performed in the Illumina RTA sequence analysis pipeline. Sequence reads were trimmed up to the first base with a quality of more than 10. Mapping to human genome build hg19 (GRCh37) was done with GEM, allowing up to 4 mismatches [9]. Reads not mapped by GEM (~4% of them) were subjected to a final round of mapping with BFAST [10]. Results were merged and only uniquely mapping non-duplicate read pairs were used for subsequent analyses. The SAMtools suite [11] with default settings was used to call SNVs and short INDELS. Variants identified in regions with low mapability [12], with a read depth of < 10 or a strand bias probability of < 0.001 were filtered out. Variants were annotated and effects predicted with ANNOVAR [13] and snpEff [14], including information from dbSNP build 135 [15], the 1000 Genomes Project [16], the Exome Variant Server (NHLBI GO Exome Sequencing Project (ESP), Seattle, WA; http://evs.gs.washington.edu/EVS/) and an internal database of sequence variants identified in a set of > 100 control samples. Tags were added for positions with high strand bias, high tail distance bias, a read depth of < 10 and those in low mapability regions. For tumour-normal comparison, the probability of a Fisher's exact test was calculated for positions with different genotypes in the two samples.

### Detection of subclonal mutations

To identify mutations present in subclonal populations inside the tumours we used a slightly different analysis pipeline. Sequence reads were aligned to the human reference genome (GRCh37) using BWA, and the alignment was consequently cleaned using SAMtools and Picard tools for mating coordinate fixing and PCR duplicate flagging. Finally, GATK indel realigner was used to realign locally around small insertion and deletions (indels). A program specifically written in-house named RAMSES (“Realignment Assisted Minimum Evidence Spotter”; Ignacio Varela, manuscript in preparation) was used to identify coordinates with a minimum value of 2, that were independently aligned with BLAT, and that gave high-quality reads reporting differences from the reference genome in the tumour sample and absolutely no evidence of the same change in the corresponding normal sample. Additionally, mutations near DNA repeats, present in the dbSNP and 1000 Genomes databases, or reported near the end of the reads, were removed. The functional consequence of the mutations was annotated using the Ensembl perl API (Ensembl database, release 69) and only coding mutations were retained.

### Secondary analysis by 454 Roche

114 candidate variants from patients 1 and 2 were validated by targeted resequencing using the GS Junior System (Roche). ~300 bp amplicons around the identified mutations were generated, to which specific adaptors were ligated (S3 Table). A pooled, barcoded mixture of amplicons was sequenced using the 454-Junior platform (Roche). The reads were aligned against the human reference genome (GRCh37) using the BWA-SW algorithm. SAMtools was used subsequently to generate bam and pileup files, which were parsed using scripts written in-house. Only those positions with a minimum coverage of 20 in both tumour and normal samples were considered. Mutations with at least 5 independent mutant reads corresponding to a minimum of 1% of the total number of reads at that position in the tumour sample, but with no mutant reads present in the corresponding normal sample, were considered to be validated.

## Supporting Information

S1 Fig Secondary analysis. Percentage of validated mutations in a selection of 92 genes from patients 1 and 2. MP (Mutational percentage): percentage of mutated reads for each mutation. MP>15%: Refers to a mutation found in 15% or more of the total number of reads in the same genomic position. Blue: Confirmed mutations; Red: Not confirmed mutations.
(TIF)

S2 Fig Distribution of validated mutations. A). Percentage of validated mutations from the secondary analysis. B) Percentage of mutations in CpG. p shows the statistical significance in Fisher´s test.
(TIF)

S1 Table Mapping and coverage metrics. tROI: Bases that are able to be captured into the genome region that is targeted in the experiment. Specificity: The percentage of non-target bp sequenced among all bases sequenced. Enrichment: Efficiency of recovery for targeted bp in relation to the efficiency of recovery for non-targeted bp, C15: percentage of bases with at least 15X coverage. Mean_cov: mean coverage of the targeted region. Median_cov: median coverage of the targeted region.
(XLS)

S2 Table Validation panel. Wt Allele: Wild type nucleotide. Mut Allele: Mutated nucleotide. Ref. Reads: Number of reads of the Wt Allele. Mut reads: Number of reads of the Wt Allele. TumorA, C, G or T: Number of reads of each nucleotide.
(XLS)

S3 Table Oligonucleotides used for validation analysis. (XLS)

S4 Table Nucleotide context in validated mutations. (XLS)

S5 Table Unique mutations (SNVs) found in this study with potential to provoke amino acid changes. Ref_base: Wild type nucleotide. Mut_base: Mutated nucleotide. Reads_A, C, G or T: Number of reads of each nucleotide. In CpG: the nucleotide is located in a CpG island. Gene ID: Gene name. Transcript ID: Transcript name. c.Annot: Mutation in the cDNA. pAnnot: Mutations in protein. Interpretation: Mutations effect.
(XLS)
