# Correction: Colorectal Adenomas Contain Multiple Somatic Mutations That Do Not Coincide with Synchronous Adenocarcinoma Specimens

**DOI:** 10.1371/journal.pone.0125459

**Published:** 2015-04-10

**Authors:** José P. Vaqué, Nerea Martínez, Ignacio Varela, Fidel Fernández, Marta Mayorga, Sophia Derdak, Sergi Beltrán, Thaidy Moreno, Carmen Almaraz, Gonzalo De las Heras, Mónica Bayés, Ivo Gut, Javier Crespo, Miguel A. Piris

An affiliation was inadvertently excluded for the first author. José P. Vaqué is also affiliated with #2 IBBTEC-UC-CSIC-SODERCAN Instituto de Biomedicina y Biotecnología de Cantabria, Santander, Spain.
