# Supplementary figures and images for: Colorectal Adenomas Contain Multiple Somatic Mutations That Do Not Coincide with Synchronous Adenocarcinoma Specimens
Source: PLoS One. 2015 Mar 16;10(3):e0119946. doi: 10.1371/journal.pone.0119946 (PMC4361059; doi:10.1371/journal.pone.0119946)

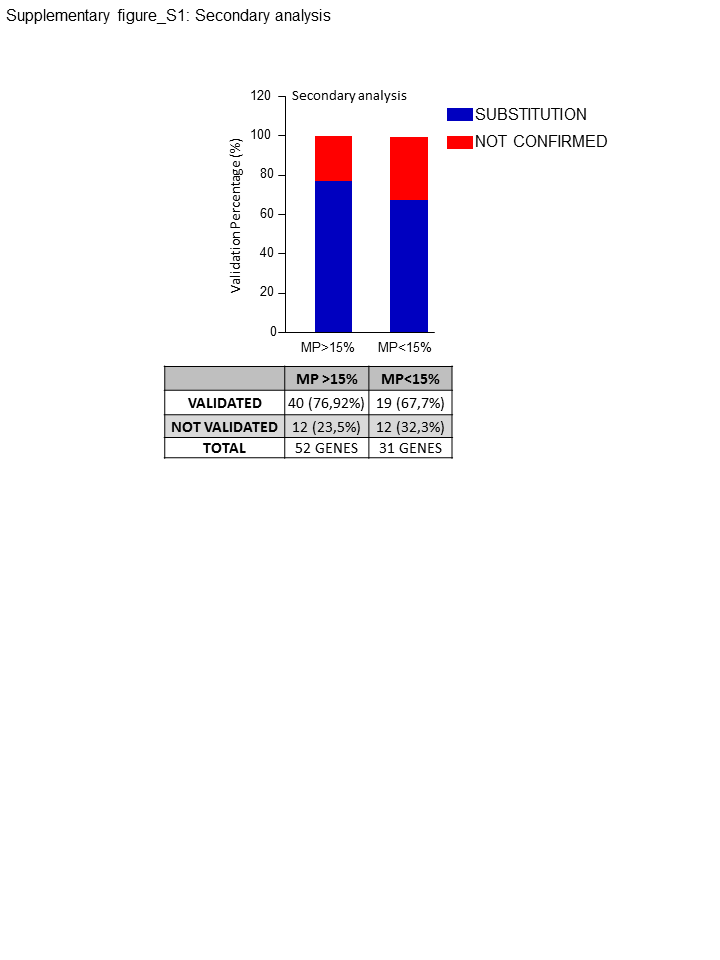

Supplement: S1 Fig — Percentage of validated mutations in a selection of 92 genes from patients 1 and 2. MP (Mutational percentage): percentage of mutated reads for each mutation. MP>15%: Refers to a mutation found in 15% or more of the total number of reads in the same genomic position. Blue: Confirmed mutations; Red: Not confirmed mutations. (TIF) [file pone.0119946.s001.tif]

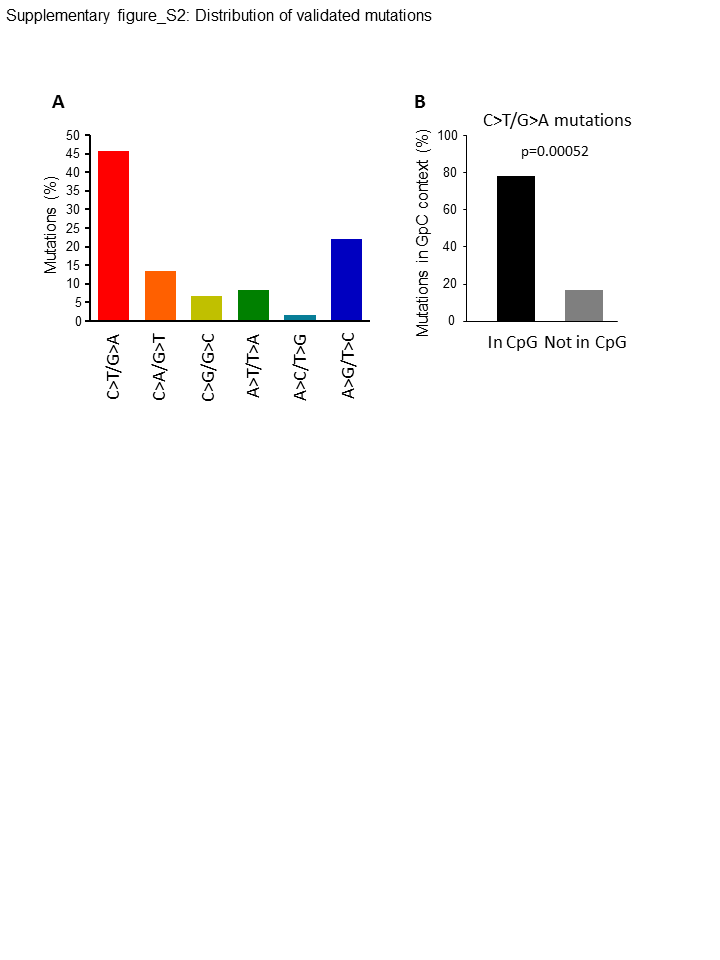

Supplement: S2 Fig — A). Percentage of validated mutations from the secondary analysis. B) Percentage of mutations in CpG. p shows the statistical significance in Fisher´s test. (TIF) [file pone.0119946.s002.tif]
